# Ultrasonic Irradiation Enables Facile Production of Lovastatin from Sugar Cane Bagasse

**DOI:** 10.1021/acsomega.1c06221

**Published:** 2022-04-12

**Authors:** Prapassorn Rugthaworn, Udomlak Sukatta, Prakit Sukyai

**Affiliations:** † Biotechnology of Biopolymers and Bioactive Compounds Special Research Unit, Department of Biotechnology, Faculty of Agro-Industry, Kasetsart University, Chatuchak, Bangkok 10900, Thailand; ‡ Kasetsart Agricultural and Agro-Industrial Product Improvement Institute (KAPI), Kasetsart University, Bangkok 10900, Thailand; § Center for Advanced Studies for Agriculture and Food, Kasetsart University Institute for Advanced Studies, Kasetsart University, Chatuchak, Bangkok 10900, Thailand

## Abstract

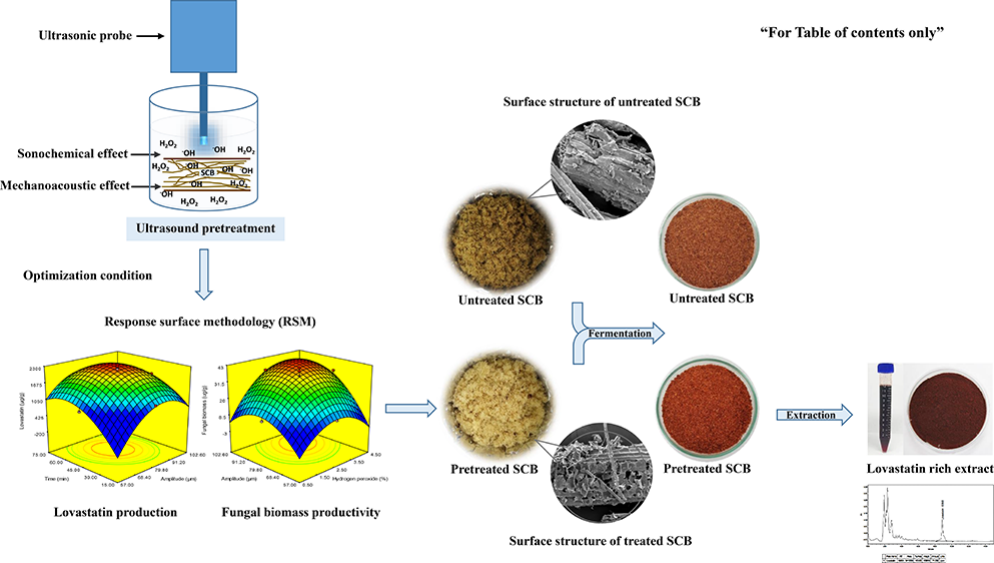

This study investigated the effect of ultrasound-assisted hydrogen peroxide (H_2_O_2_) pretreatment on sugar cane bagasse (SCB) followed by *Monascus purpureus* TISTR 3003 cultivation for lovastatin production under solid-state fermentation (SSF). Optimization of the pretreatment conditions was investigated using a response surface methodology (RSM). Within the range of the selected operating conditions, the optimized values of H_2_O_2_ concentration, amplitude, SCB dosage, and sonication time were found to be 2.74%, 83.22 μm, 2.84% and 52.29 min, respectively. The *R*^2^ value of 0.9749 indicated that the fitted model is in good agreement with the predicted and actual lovastatin production. On the basis of the optimum conditions, the lovastatin production was 2347.10 ± 17.19 μg/g, which is 2.4 times higher than that under untreated conditions. Scanning electron microscopy (SEM) analysis explored the surface structure of the untreated SCB, which showed a compact rigid structure. In contrast, treated SCB had a rough surface structure and cracks as a result of the pretreatment.

## Introduction

Lovastatin is a potent competitive inhibitor of the rate-limiting enzyme in the cholesterol biosynthesis of 3-hydroxy-3-methyl glutaryl coenzyme A (HMG-CoA) reductase.^1^ Lovastatin can be produced from numerous fungi such as *Penicillium* spp.,^2^*Aspergillus terreus*,^3^ and *Monascus purpureus*.^4^*M. purpureus* is a nonpathogenic fungus which has been used traditionally in China and Japan for natural pigment production along with lovastatin under solid-state fermentation (SSF).^5^ Different substrates have been used for lovastatin production through SSF, including sorghum grain and wheat bran^6^ and rice.^4^ However, considering the abundance and easy accessibility of lignocellulosic waste such as sugar case bagasse (SCB) in Thailand and our research expertise, we have the conviction that SCB would be a promising substrate for lovastatin production. It is mainly composed of cellulose, which is rich in sugar fractions and can be broken down as a carbon source.^7^ Hilares et al.^8^ and other scholars investigated SCB as a substrate for pigment production by *Monascus* sp. Similarly, Lu et al.^9^ have reported SCB as a carrier of the medium in the conversion of glycerol to lovastatin under SSF. Despite the various studies, there has been no report on the direct use of SCB as a lignocellulosic substrate for lovastatin production. This may be due to its recalcitrant lignocellulose structure. Therefore, a pretreatment of SCB to enhance the accessibility of its surface area and porosity is required prior to SSF.

Pretreatment of lignocellulose has been an actively researched field for several decades. However, many of these approaches are energy-consuming and employ chemicals which require special disposal and handling methods.^10^ Thus, ultrasonic pretreatment is an emerging green technology that has a high efficiency for the structural modification of lignocellulose. The ultrasound produces mechanoacoustic and sonochemical effects,^11^ which promotes lignocellulose deconstruction.^12^ Over the years, ultrasound has been viewed as a viable option, coupled with other chemicals, to enhance the efficacy of pretreatment. Xu et al.^13^ studied an ultrasound-assisted aqueous ammonia pretreatment for the intensification of the enzymatic hydrolysis of corn cobs and found that the ammonia concentration, solid content, and ultrasonication time have an effect on lignin removal. Ramadoss and Muthukumar^14^ reported that the ultrasound-assisted H_2_O_2_ pretreatment of SCB increased the delignification capability from 35.7% to 38.17% in comparison with ultrasound alone. They also studied the effect of ultrasound pretreatment of SCB using a metal salt (titanium dioxide) with H_2_O_2_ and found that the rate and yield of hydrolysis were enhanced by the process. However, metal salts and chemicals as well as environmental conditions affect the growth of microorganisms. Therefore, this study used only ultrasound and H_2_O_2_ as a pretreatment process due to their advantages such as operation under mild conditions with less contamination of hazardous chemicals in the biomass.

To the best of our knowledge, no study has been conducted regarding the effect of ultrasound combined with H_2_O_2_ on the pretreatment of SCB as a lignocellulosic substrate for improving lovastatin production by *Monascus purpureus* TISTR 3003 under SSF. Thus, this research was performed according to central composite design (CCD) and response surface methodology (RSM) to optimize the ultrasound-assisted H_2_O_2_ pretreatment factors such as H_2_O_2_ concentration, amplitude, SCB dosage, and sonication time for a better substrate preparation and subsequent fermentation process.

## Results and Discussion

### Analysis of Variance and Model Fitting

Our studies used ultrasound-assisted H_2_O_2_ pretreatment of SCB using RSM to identify the optimum key factors influencing lovastatin production by *M. purpureus* TISTR 3003 under SSF. The effects of four factors (H_2_O_2_ concentration, amplitude, SCB dosage, and sonication time) were explored by CCD. An experimental design of 27 runs consisted of 16 factorial points, 3 center points, and 8 axial points. The consequent range of the experiment and the coded levels (−α, −1, 0, 1, α; α = 2) are given in Table 1. Table 1 shows the results of actual and predicted data of lovastatin production and fungal biomass by a quadratic model. The optimum conditions (2.5% H_2_O_2_, amplitude 79.8 μm, 3% SCB dosage, and sonication time of 45 min) resulted in a maximum lovastatin production of 2295 μg/g, as shown in run order 27. Moreover, our study showed that lovastatin production related to the productivity of fungal biomass was 39.08 μg/g in run order 27. The statistical significance of the model (eq 1) of lovastatin yield checked by an *F* test and an analysis of variance (ANOVA) of the response surface quadratic model are given in Table 2, whereas the model (eq 2) of the fungal biomass productivity is statistically significant; the response surface quadratic model is given in Table 3. An ANOVA of the regression model for lovastatin production gave an *F* value of 33.34 and a *P* value of less than 0.05, which implies that the model is significant. Likewise, the ANOVA of the regression model of fungal biomass yield showed an *F* value of 46.36 and a *P* value of less than 0.05, which indicates that the model is significant.

**Table 1:** Central Composite Design Matrix of Four Variables with Actual and Predicted Response Values

	level				actual level				lovastatin (μg/g)		fungal biomass (μg/g)	
run order	*X*_1_	*X*_2_	*X*_3_	*X*_4_	H_2_O_2_ (%)	amp (μm)	SCB (%)	time (min)	actual	predicted	actual	predicted
1	–1	–1	–1	–1	1.5	68.4	2	30	990	930	8.73	8.16
2	1	–1	–1	–1	3.5	68.4	2	30	1131	1179	10.02	12.96
3	–1	1	–1	–1	1.5	91.2	2	30	1294	1266	22.35	22.66
4	1	1	–1	–1	3.5	91.2	2	30	1427	1515	32.73	34.18
5	–1	–1	1	–1	1.5	68.4	4	30	949	833	8.33	5.72
6	1	–1	1	–1	3.5	68.4	4	30	1083	1082	11.36	10.52
7	–1	1	1	–1	1.5	91.2	4	30	1109	1169	19.82	20.22
8	1	1	1	–1	3.5	91.2	4	30	1378	1418	33.71	31.74
9	–1	–1	–1	1	1.5	68.4	2	60	1347	1393	32.13	29.72
10	1	–1	–1	1	3.5	68.4	2	60	1655	1642	35.29	34.52
11	–1	1	–1	1	1.5	91.2	2	60	1522	1458	32.13	28.02
12	1	1	–1	1	3.5	91.2	2	60	1764	1707	36.66	39.54
13	–1	–1	1	1	1.5	68.4	4	60	1344	1296	25.35	27.28
14	1	–1	1	1	3.5	68.4	4	60	1709	1545	32.29	32.08
15	–1	1	1	1	1.5	91.2	4	60	1258	1362	24.56	25.58
16	1	1	1	1	3.5	91.2	4	60	1484	1611	37.13	37.10
17	–2	0	0	0	0.5	79.8	3	45	1015	1063	12.56	15.24
18	2	0	0	0	4.5	79.8	3	45	1600	1561	33.66	31.56
19	0	–2	0	0	2.5	57	3	45	1015	1164	13.19	14.08
20	0	2	0	0	2.5	102.6	3	45	1705	1566	33.92	33.60
21	0	0	–2	0	2.5	79.8	1	45	1208	1224	25.82	25.60
22	0	0	2	0	2.5	79.8	5	45	1036	1030	19.88	20.72
23	0	0	0	–2	2.5	79.8	3	15	1239	1219	16.03	16.14
24	0	0	0	2	2.5	79.8	3	75	1845	1875	42.55	43.06
25	0	0	0	0	2.5	79.8	3	45	2265	2252	37.44	38.60
26	0	0	0	0	2.5	79.8	3	45	2197	2252	41.29	38.60
27	0	0	0	0	2.5	79.8	3	45	2295	2252	39.08	38.60

**Table 2:** Analysis of Variance (ANOVA) Results for Lovastatin Concentration^a^

source	sum of squares	df	mean square	*F*	*p*	comment
model	3.849 × 10^006^	14	2.749 × 10^005^	33.34	0.0001**	significant
*X*_1_ (H_2_O_2_ conc)	3.720 × 10^005^	1	3.720 × 10^005^	45.11	<0.0001**	
*X*_2_ (amplitude)	2.416 × 10^005^	1	2.416 × 10^005^	29.30	0.0002**	
*X*_3_ (SCB dosage)	56066.67	1	56066.67	6.80	0.0229**	
*X*_4_ (time)	6.448 × 10^005^	1	6.448 × 10^005^	78.20	<0.0001**	
*X*_1_*X*_2_	380.25	1	380.25	0.046	0.8336	
*X*_1_*X*_3_	1806.25	1	1806.25	0.22	0.6481	
*X*_1_*X*_4_	13456.00	1	13456.00	1.63	0.2256	
*X*_2_*X*_3_	34225.00	1	34225.00	4.15	0.0643	
*X*_2_*X*_4_	73170.25	1	73170.25	8.87	0.0115**	
*X*_3_*X*_4_	1806.25	1	1806.25	0.22	0.6481	
*X*_1_^2^	1.178 × 10^006^	1	1.178 × 10^006^	142.85	<0.0001**	
*X*_2_^2^	1.050 × 10^006^	1	1.050 × 10^006^	127.34	<0.0001**	
*X*_3_^2^	1.689 × 10^006^	1	1.689 × 10^006^	204.80	<0.0001**	
*X*_4_^2^	6.635 × 10^005^	1	6.635 × 10^005^	80.46	<0.0001**	
residual	98948.75	12	8245.73			
lack of fit	93906.08	10	9390.61	3.72	0.2301	not significant
total	3.948 × 10^006^	26				

std dev	CV (%)	PRESS	sdeq precision	*R*^2^	adj *R*^*2*^	pred *R*^*2*^
90.81	6.31	5.522 × 10^005^	19.929	0.9749	0.9457	0.8601

a ** denotes significance <0.01, and * denotes significance <0.05.

**Table 3:** Analysis of Variance (ANOVA) Results for Productivity of Fungal Biomass^a^

source	sum of squares	df	mean square	*F*	*p*	comment
model	2985.89	14	213.28	46.36	<0.0001**	significant
*X*_1_ (H_2_O_2_ conc)	400.09	1	400.091	86.97	<0.0001**	
*X*_2_ (amplitude)	570.86	1	570.86	124.09	<0.0001**	
*X*_3_ (SCB dosage)	35.94	1	35.94	7.81	0.0162**	
*X*_4_ (time)	1087.16	1	1087.16	236.32	<0.0001**	
*X*_1_*X*_2_	45.39	1	45.39	9.87	0.0085**	
*X*_1_*X*_3_	18.21	1	18.21	3.96	0.0699	
*X*_1_*X*_4_	0.12	1	0.12	0.026	0.8740	
*X*_2_*X*_3_	2.256 × 10^–003^	1	2.256 × 10^–003^	4.904 × 10^–004^	0.9827	
*X*_2_*X*_4_	262.04	1	262.04	56.96	<0.0001**	
*X*_3_*X*_4_	16.54	1	16.54	3.60	0.0822	
*X*_1_^2^	307.50	1	307.50	66.84	<0.0001**	
*X*_2_^2^	289.74	1	289.741	62.98	<0.0001**	
*X*_3_^2^	318.12	1	318.12	69.15	<0.0001**	
*X*_4_^2^	108.15	1	108.15	23.51	0.0004**	
residual	55.21	12	4.60			
lack of fit	37.75	10	3.78	0.43	0.8504	not significant
total	3041.09	26				

std dev	CV (%)	PRESS	adeq precision	*R*^2^	adj *R*^2^	pred *R*^2^
2.14	8.09	256.73	23.441	0.9818	0.9607	0.9156

a ** denotes significance <0.01, and * denotes sgnificant <0.05.

The coefficient of variation (CV) is a useful statistic for expressing the degree of precision. A lower value of CV is related to a greater precision of the results; thus, a CV value lower than 10% means that the experiment has high precision.^15^ The results of lovastatin production and fungal biomass productivity gave CV values of 6.31% and 8.09%, respectively, which demonstrate a good model with a high quality of prediction. The accuracy of a model can be evaluated by determining the coefficient (*R*^2^). A regression model having an *R*^2^ value close to 1 implies a strong correlation between the experimental results and the theoretical values predicted by the model equation.^15,16^

The *R*^2^ value in this study suggests that the sample variation of lovastatin production and the productivity of biomass are 0.9749 and 0.9818, respectively, indicating a close agreement between the experimental results and the predicted theoretical values. The quadratic model equation of lovastatin production showed high values of the predicted *R*^2^ and the adjusted *R*^2^ of 0.8601 and 0.9457, respectively, with the difference being less than 0.2. However, the model for fungal biomass also showed high values of the predicted *R*^2^ and the adjusted *R*^2^ of 0.9156 and 0.9607, respectively. Furthermore, a model with a signal to noise ratio greater than 4 is desirable in an adequate model.^16^ The signal to noise ratios recorded for lovastatin production and fungal biomass yield in this study are 19.929 and 23.441, respectively, demonstrating appropriate signals for this model. The *F* values of the lack of fit for lovastatin production and productivity of the biomass are 3.72 and 0.43, respectively, which suggest that the lack of fit is not significant. A nonsignificant lack of fit is an indication that the linear regression is an adequate model to describe the effect of the pretreatment of SCB for lovastatin production and productivity of fungal biomass. The results indicated that both dependent variables, lovastatin production and fungal biomass yield, were positively correlated, in which an increasing fungal biomass productivity expressed the high growth rate of *M. purpureus* that affects the production of lovastatin. By an analysis of the experimental data, a second-order polynomial model equation of the four variables for lovastatin yield as a reduced form of equation in significant term of coded factors is
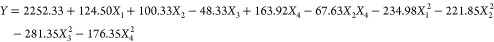
1where *Y* is the lovastatin concentration (μg/g) and *X*_1_, *X*_2_, *X*_3_, and *X*_4_ are code values of H_2_O_2_ concentration (%), amplitude (μm), SCB dosage (%), and sonication time (min), respectively.

Regression analysis was performed on the results of fungal biomass as the independent variables, and the second-order polynomial equation was derived as eq 2
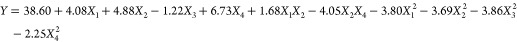
2where *Y* is the productivity of fungal biomass (μg/g) and *X*_1_, *X*_2_, *X*_3_, and *X*_4_ are code values as described above.

Tables 2 and 3 give an estimation of the parameters, and the corresponding *P* values of the factors *X*_1_*, X*_2_*, X*_3_, and *X*_4_ are significant terms. Positive coefficients for *X*_1_ (H_2_O_2_ concentration), *X*_2_ (amplitude), and *X*_4_ (sonication time) indicated a linear effect to increase lovastatin concentration and fungal biomass, while the negative coefficient of *X*_3_ (SCB dosage) revealed an opposite effect on lovastatin yield and the productivity of fungal biomass, as shown in eqs 1 and 2, respectively. Meanwhile, the interaction between amplitude (*X*_2_) and sonication time (*X*_4_) was found to be significant, as the model Prob > *F* is less than 0.05 for lovastatin production and fungal biomass, as shown in Tables 2 and 3. The interaction between H_2_O_2_ concentration (*X*_1_) and amplitude (*X*_2_) was found to be significant only for the productivity of fungal biomass, as shown in Table 3. However, the interactions between the other pairs of variables were found to be insignificant for lovastatin production and fungal biomass content.

This result is similar to the study by Ramadoss and Muthukumar,^14^ who evaluated ultrasound-assisted pretreatment of SCB using a metal salt with H_2_O_2_ for cellulose recovery. They found that the concentrations of H_2_O_2_ and biomass, ultrasonication time, molar ratio of metal salts to H_2_O_2_, temperature, amplitude, and ultrasound duty cycle all had effects on the delignification process of SCB. Likewise, our work showed that lovastatin production from SCB is affected by the operating conditions, including H_2_O_2_ concentration, amplitude, SCB dosage, and sonication time.

### Diagnostics and Adequacy of the Model on Lovastatin Production

**Figure 1:**
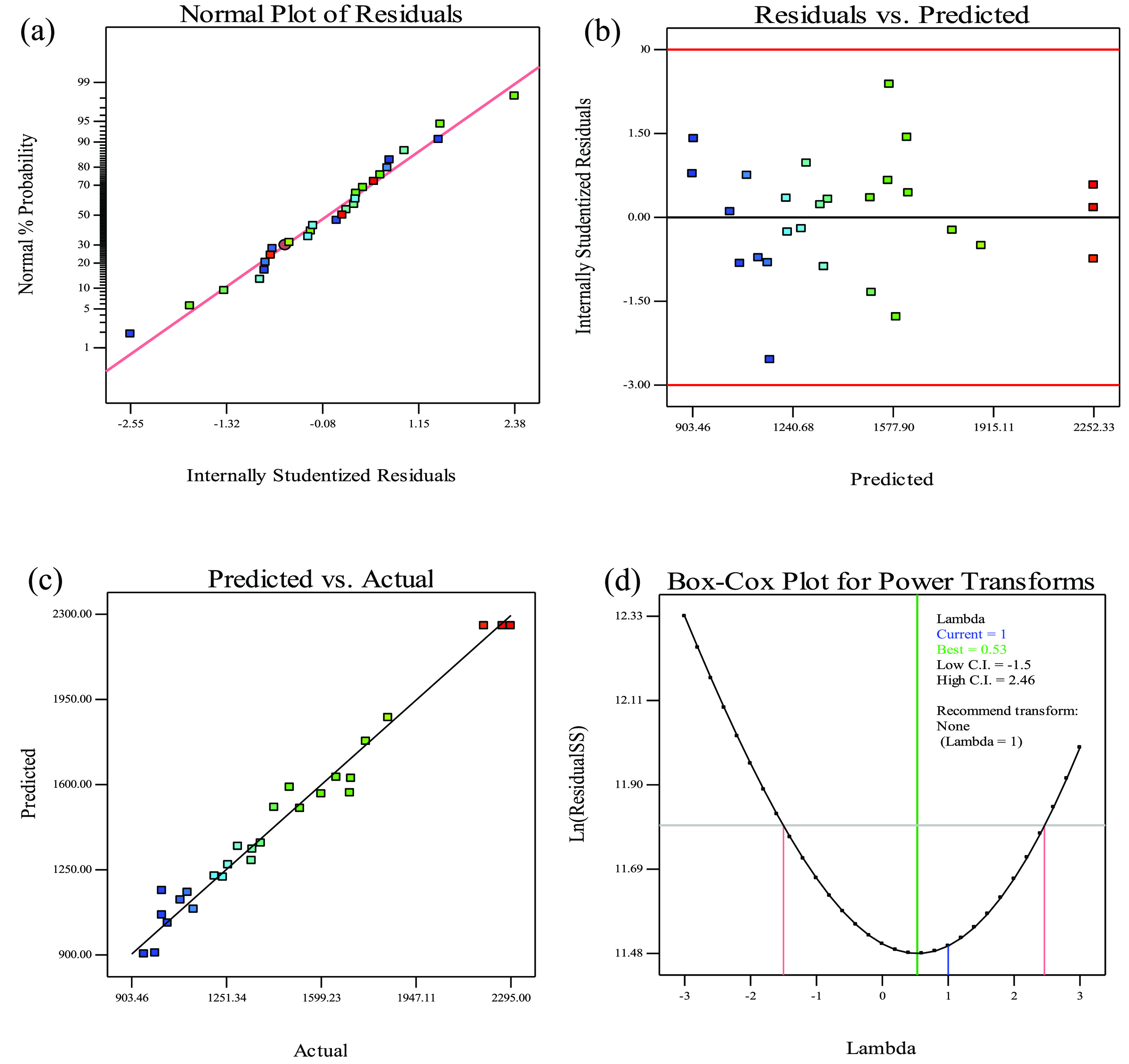
Diagnostics and adequacy of the model for the response (recovery) shown by (a) normal probability plot of Studentized residuals, (b) a plot of internally Studentized residuals vs predicted response, (c) a diagnostic plot of the model precision, and (d) Box-Cox plot of model transformation.

It is essential to evaluate the adequacy of the model to see if it is able to represent the experimental values, before investigating the interaction between the variables and their effect on the optimization process. In this analysis, the internally Studentized residuals are linear (Figure 1a), which indicated that the residuals followed a normal distribution. The adequacy of the model was also examined by plotting residuals against the predicted response which are randomly scattered around the zero line (Figure 1b). This pattern showed good data distribution indicating that the residuals follow a normal distribution. The normality assumption was further confirmed by the residual plot, which presented a straight line (Figure 1c), demonstrating that the actual data are consistent with the predicted values. Thus, all diagnostic plots confirmed that the model is adequate. The Box-Cox plot showed minimum and maximum confidence interval (CI) values of −1.5 and 2.46, respectively (Figure 1d). The natural logarithm (ln) of the residual sum of squares (SS) against λ was 1 and decreased suddenly with a minimum value in the region of 0.53 (Figure 1d). The experimental data did not require transformation, as the current value of CI (λ) was close to the optimum value.^16,17^ Hence, this verified the spread of data and appropriateness of the proposed model for pretreatment of SCB as a substrate of SSF for lovastatin production. The adequacy of the overall analysis signified that the selected CCD model is accurate and reliable.

**Figure 2:**
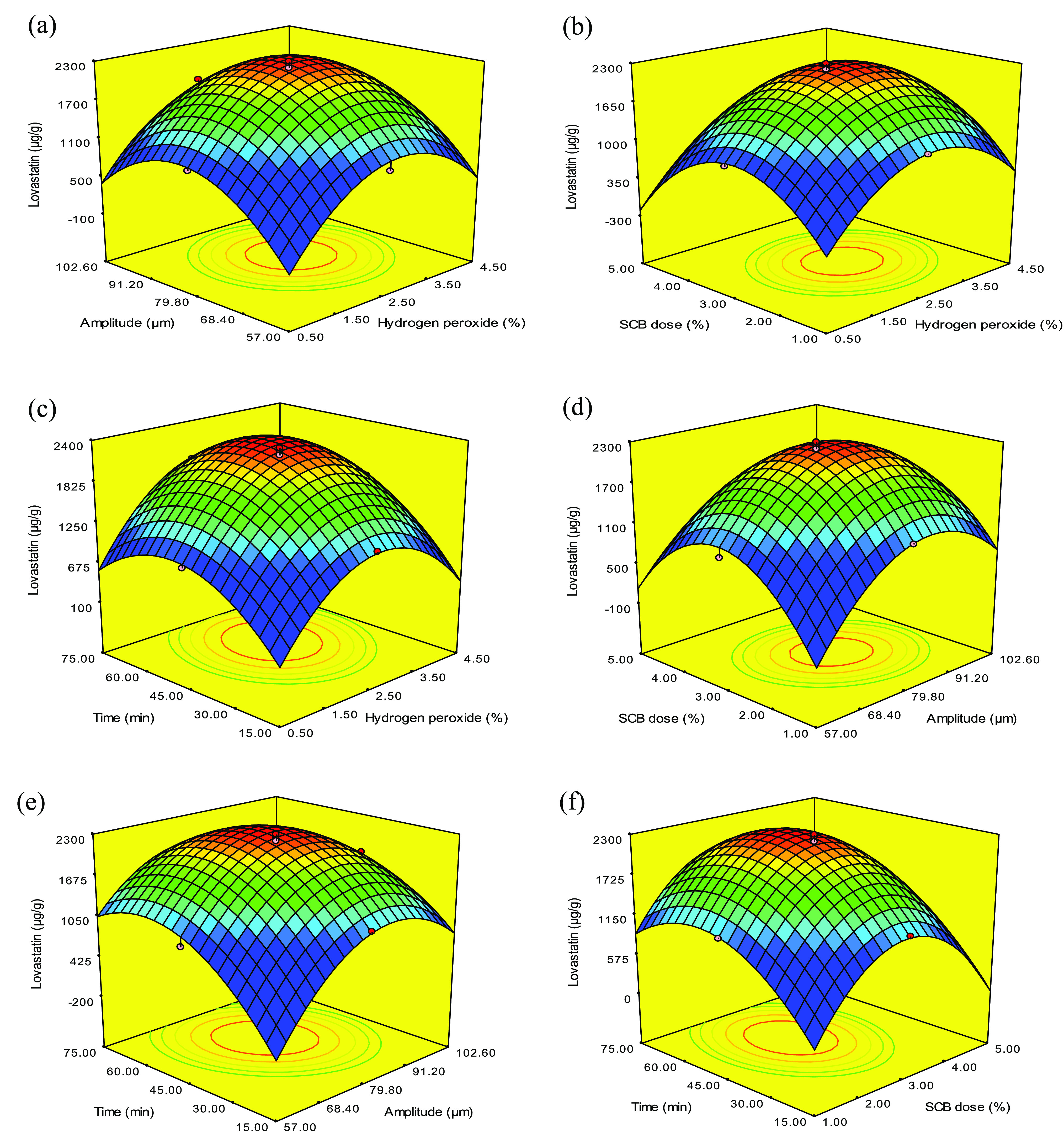
Response surface graph for the effects of the (a) H_2_O_2_ concentration (%) versus amplitude (μm), (b) H_2_O_2_ concentration (%) versus SCB dosage (%), (c) H_2_O_2_ concentration (%) versus reaction time (min), (d) amplitude (μm) versus SCB dosage (%), (e) reaction time (min) versus amplitude (μm), and (f) SCB dosage (%) versus reaction time (min) on the yield of lovastatin.

Three-dimensional responses generated for the pairwise combination of the four factors for lovastatin production are shown in Figure 2, which depicts the relationship between the two chosen variables. Lovastatin production increased with increasing H_2_O_2_ concentration and amplitude up to 2.5% (Figure 2a–c) and 79.8 μm (Figure 2a,d,e), respectively. Ultrasound promoted the dissociation of H_2_O_2_ into hydroxyl radicals, which stimulated the deconstruction of lignin and hemicellulose.^18^ This relates to the ability of the microorganisms to use SCB as a substrate for growth^19^ and produce fungal metabolites.^20^ However, H_2_O_2_ itself acts as a hydroxyl radical “scavenger”, which decreases the rate of delignification during the oxidation of lignin.^21^ A lower concentration of H_2_O_2_ did not form sufficient hydroxyl radicals, thus making the process ineffective. Therefore, finding the optimization concentration of H_2_O_2_ is very important. In the case of ultrasound, the amplitude is an important parameter and it is related to the power of the ultrasound beam. Higher amplitudes may present an undesirable effect by reducing the deconstruction of the compact structure of SCB. The presence of numerous explosion bubbles near the end of the probe may impede the energy flow from the probe to the solution.^22^ Hence, 79.8 μm was determined as the optimum amplitude.

Similarly, with an increase in SCB dosage to 3% the lovastatin production increased, but a further increase in dosage did not increase the yield of lovastatin (Figure 2b,d,f). A higher concentration of solids might increase the viscosity while reducing mixing as well as mass and heat transfer.^23^ In contrast, at a lower dose of substrate the presence of radicals might solubilize part of the cellulosic and hemicellulosic fractions.^24^ Hence, the biomass concentration that will be appropriate for increasing cavitation activity while decreasing mass and heat transfer phenomena should be considered.^25^

In the case of sonication time, as shown in Figure 2c,e,f, lovastatin production increased with an increase in sonication time to 60 min. This may be due to increased effects of cavitation that enhanced the rates of reactions.^26^ The sonication time required depends on the amplitude. The mutual interaction between amplitude (*X*_2_) and sonication time (*X*_4_) was found to be significant, as the model Prob > *F* is less than 0.05, as shown in Figure 2e. The pretreatment time for SCB was shortened from 60 to 45 min by increasing the amplitude from 68.4 to 102.6 μm for high lovastatin production (1705–2295 μg/g). A similar trend was reported by Kunaver et al.^27^ for the absolute liquefaction of spruce wood meal by ultrasound. The liquefaction time was reduced from 80 to 10 min by increasing the amplitude from 20% to 100%.

### Validation of the Model

The result of the optimization process through RSM was validated by performing several experiments generated by Design Expert software and comparing the actual and predicted outcomes, as shown in Table 4. The optimum values were determined as 2.74%, 83.22 μm, 2.84%, and 52.29 min for H_2_O_2_ concentration, amplitude, SCB dosage, and sonication time, respectively. These values predicted the production of lovastatin to be 2312.73 μg/g. The optimum conditions were verified in a triplicate solid-state study, and an average production of 2347.10 ± 17.19 μg/g of lovastatin was observed. The experimental results were in good agreement with the predicted responses, indicating that the model is valid. The predicted response matched well with the experimental data, showing the validity of the optimization process. The potential of ultrasound-assisted H_2_O_2_ pretreatment of SCB in lovastatin production was proved by subsequent experiments.

**Table 4:** Optimized Ultrasound-Assisted Pretreatment Conditions and Lovastatin Production

	actual level				lovastatin (μg/g)	
no.	H_2_O_2_ (%)	amp (μm)	SCB (%)	time (min)	actual	predicted
1	2.62	82.08	2.90	48.90	2297.70 ± 16.00	2306.30
2	2.64	79.8	2.92	56.43	2275.63 ± 26.26	2295.08
3	2.83	82.08	2.64	51.15	2285.91 ± 22.66	2299.04
4	2.80	80.94	2.88	51.71	2301.45 ± 26.08	2317.74
5	2.74	83.22	2.84	52.29	2347.10 ± 17.19	2312.73
6	2.55	79.8	2.89	51.87	2235.34 ± 13.56	2297.20

### Effect of Pretreatment on Fungal Growth

Table 5 gives the lovastatin content and fungal biomass yield of *M. purpureus* TISTR 3003 on untreated and treated SCB after fermentation for 20 days. The cultivation of *M. purpureus* TISTR 3003 on the treated SCB showed a lovastatin content of 2347.10 ± 17.19 μg/g, which is 2.4 times higher than that for untreated SCB (977.00 ± 35.91 μg/g). The experimental data suggest that ultrasound pretreatment is a valuable tool in the preparation of SCB as a substrate for enhanced lovastatin production. Likewise, our study showed that lovastatin production is related to the ability of fungi to grow on the substrate. The maximum fungal biomass yield of 42.39 μg/g cell dry weight was obtained from the cultivation of *M. purpureus* TISTR 3003 on the treated SCB under the optimized conditions (Table 5). This might be due to breaking down of the surface structure of SCB by the pretreatment process, thereby increasing the porosity and available surface area of SCB, which supported the growth of fungi.^19,28^ The lovastatin concentration increased with an increase in fungal biomass yield; this result is similar to that in a previous experiment. A similar trend was reported by Dhar and Nigam,^29^ where the maximum lovastatin production was obtained at the highest fungal biomass yield of *A. terreus* after cultivation for 7 days.

**Table 5:** Comparison of the Production of Lovastatin and Fungal Biomass Yield of Native and Treated SCB after Fermentation

SCB	lovastatin^a^ (μg/g)	fungal biomass^a^ (μg/g cell dry weight)
native	977.00 ± 35.91	22.29 ± 0.45
treated	2347.10 ± 17.19	42.39 ± 0.03

a *p* <0.01 parameters between native and treated SCB (independent *t* test).

### Morphological Characteristics of SCB

**Figure 3:**
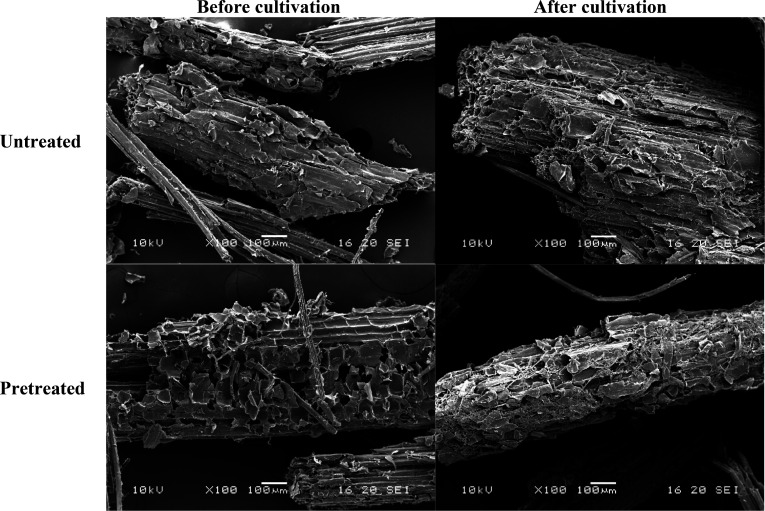
SEM images of untreated and pretreated SCB samples before and after fermentation for 20 days.

In this study, SEM was used to explore the surface structure of lignocellulosic SCB. Figure 3 shows the SEM micrographs of untreated and treated SCB samples. Before fermentation, the untreated SCB has a dense structure with a smooth and unbroken surface. In contrast, the surface structure of treated SCB was broken into a very rough surface by the pretreatment. This effect indicated that the cross-linking among cellulose, hemicellulose, and lignin was interrupted by pretreatment, which resulted in the separation of some fibers.^24^ Similar reports by Wu et al.^12^ and Xu et al.^13^ indicated that the resulting microjets and hydroxyl radicals produced during the collapse of the bubbles could break the interior and surface of corncobs in close proximity, resulting in the decomposition of lignocellulose.^30^ The fragile structure of treated SCB suggests a large surface that is accessible to the enzymes produced by the microorganism and an increase in fungal growth. This explained the high density of fungal mycelium on the surface of treated SCB in comparison with that of untreated SCB after cultivation (Figure 3), which was reflected in the lovastatin concentration obtained.

### Chemical Composition of SCB

The chemical compositions of untreated and treated SCB samples are presented in Table 6. Before fermentation, the untreated SCB had a lignin content of approximately 20.94 ± 0.16% in combination with hemicellulose (31.96 ± 1.36%) and cellulose (40.83 ± 0.40%). After pretreatment, the lignin and hemicellulose contents decreased to 18.44 ± 0.24% and 30.85 ± 0.35%, respectively, which may be due to the disintegration of carbohydrate–lignin linkages by ultrasound.^31^ The cellulose content of treated SCB increased to 42.74 ± 1.78% before fermentation and decreased to 39.30 ± 0.49% after fermentation for 20 days. The decrease could be associated with the ability of microorganisms to use the cellulose, which is rich in sugar fractions and can be broken down as a carbon source for the growth and production of fungal metabolites.^20^

**Table 6:** Effect of Pretreatment on Chemical Composition of SCB Biomass

	before fermentation			after fermentation		
SCB	lignin^a^ (%)	hemicellulose^ns^ (%)^b^	α-cellulose^ns^ (%)^b^	lignin^a^ (%)	hemicellulose^ns^ (%)^b^	α-cellulose^ns^ (%)^b^
native	20.94 ± 0.16	31.96 ± 1.36	40.83 ± 0.40	21.34 ± 0.38	29.08 ± 0.59	39.23 ± 0.51
treated	18.44 ± 0.24	30.85 ± 0.35	42.74 ± 1.78	18.64 ± 0.11	27.79 ± 0.47	39.30 ± 0.49

a *p* < 0.01 parameters between native and treated SCB (independent *t* test).

b ns not significant.

## Conclusion

The present study demonstrated the successful production of lovastatin by *M. purpureus* TISTR 3003 under SSF using ultrasound-assisted H_2_O_2_ pretreatment of SCB as a lignocellulosic substrate. The maximum lovastatin concentration was achieved by employing an H_2_O_2_ concentration of 2.74%, an amplitude of 83.22 μm, an SCB dosage of 2.84%, and a sonication time of 52.29 min as the optimum conditions. Under the optimum conditions, the lovastatin content was 2347.10 ± 17.19 μg/g, which is much higher than that in untreated SCB. The result indicated that applying RSM as an optimization technique can increase the potential of an ultrasound pretreatment in the substrate preparation process for SSF.

## Experimental Section

### Materials

SCB was obtained from Khonburi Sugar Public Company Limited Factory, Nakhon Ratchasima province, Thailand. *Monascus purpureus* TISTR 3003 was obtained from the Microbiological Resources Centre, Thailand Institute of Scientific and Technological Research (TISTR), Thailand. Lovastatin and *N*-acetylglucosamine were obtained from Sigma-Aldrich (USA). All other reagents used were of analytical grade and were used as received without further purification.

### Microorganisms and Culturing Conditions

A spore suspension containing 10^8^ spores/mL of *M. purpureus* TISTR 3003 was inoculated into a 500 mL Erlenmeyer flask containing 100 mL of culture medium. The components of the culture medium were glucose (60 g/L), peptone (25 g/L), NaNO_3_ (2 g/L), MgSO_4_·7H_2_O (1 g/L), and KH_2_PO_4_ (1 g/L). The inoculum culture was incubated at 30 °C for 2 days with shaking at 170 rpm.

### Procedure of Solid-State Fermentation

SCB was used as the lignocellulosic substrate for lovastatin production in SSF. Briefly, 5 g of untreated and treated SCB from the various pretreatment conditions were placed separately in 500 mL Erlenmeyer flasks and moistened with distilled water containing 200 g/L of glycerol, 100 g/L of soybean powder, 10 g/L of NaNO_3,_ 5 g/L of MgSO_4_·7H_2_O, 5 g/L of K_2_HPO_4_3·H_2_O, 10 g/L of ZnSO_4_7·H_2_O, and 50 mL/L of corn steep liquor to maintain a moisture content of 60% (v/w). The substrates were sterilized at 121 °C for 20 min. After cooling, 10% (v/w) inoculum was cultured on the substrate and incubated at 30 °C for 3 days followed by cultivation at 25 °C for 17 days. Three flasks as biological triplicates were used for an analysis of lovastatin concentration and biomass. After cultivation, the native and pretreated SCBs were dried at 60 °C for 48 h before the determination of lovastatin, fungal biomass, and chemical composition for a comparison of the effect of the pretreatment process on lovastatin production and the chemical composition of SCB.

### Optimization of Pretreatment Conditions

#### Experimental Design

RSM was used to determine the variables and response data. The influence of operating parameters such as H_2_O_2_ dosage (*X*_1_) in the range from 0.5 to 4.5% (v/v), ultrasound amplitude (*X*_2_) in the range from 57 to 102.6 μm, SCB dosage (*X*_3_) in the range from 1 to 5% (w/v), and sonication time (*X*_4_) in the range from 15 to 75 min was investigated for their effect on the pretreatment of SCB for maximum lovastatin production. CCD was used to evaluate the experimental parameters.^32^ A four-factor experimental matrix was developed in Design-Expert software (Stat-Ease Inc., Minneapolis, MN, USA, ver. 7.0.0). All of the experiments were done in triplicate, and the average of the lovastatin and fungal biomass yield obtained were taken as the response (*Y*). The second-order polynomial model predicting the level of lovastatin production and productivity of fungal biomass are expressed as eq 3
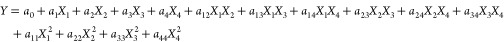
3where *Y* is the predicted response yields of lovastatin (μg/g) and the productivity of fungal biomass (μg/g), *a*_0_ is a constant coefficient, *a*_1_, *a*_2_, *a*_3_, and *a*_4_ are linear coefficients, *a*_11_, *a*_22_, *a*_33_, and *a*_44_ are quadratic coefficients, *a*_12_, *a*_13_, *a*_14_, *a*_23_, *a*_24_, and *a*_34_ are second-order interaction coefficients of the model, and *X*_1_, *X*_2_, *X*_3_, and *X*_4_ are independent variables.

The statistical analysis of the data was carried out using SPSS statistics software (version 17.0; IBM).

#### Pretreatment Process

The SCB with particle size of 1.2–1.6 mm was pretreated using an ultrasound-assisted H_2_O_2_ pretreatment. The pretreatment used a titanium probe type sonolyzer (Sonics & Materials, Inc., Model VCX 750, USA) with a 13 mm diameter probe operating at a frequency and power of 20 kHz and 750 W, respectively. The SCB was initially dispersed in 200 mL of H_2_O_2_ at the desired concentrations from 0.5% to 4.5% (v/v) in an Erlenmeyer flask. The contents were subjected to ultrasonic radiation in order to break up the SCB structure under various operating conditions.

### Analytical Methods

#### Lovastatin Analysis

Lovastatin was extracted from 0.5 g of dry fermented SCB with 50 mL of 70% (v/v) ethanol at 55 °C for 60 min. The quantitative analysis of lovastatin was carried out by high-performance liquid chromatography (HPLC, Shimadzu, Japan) using the method described by Kamath et al.^33^ In brief, a Supelco C18 column (4.6 × 250 mm, 5 μm) and a mobile phase of acetonitrile/water (70/30 (v/v)) was acidified with orthophosphoric acid to a concentration of 1.1% and a flow rate of 1.5 mL/min was used. Detection was carried out by a UV detector at 238 nm with an injection volume of 20 μL.

#### Fungal Biomass Estimation

The fungal biomass was evaluated by a determination of the quantity of *N*-acetylglucosamine. A 1 g portion of the dried culture was rinsed with 50 mL of 5 M H_2_SO_4_ for 15 min. The sediment was washed twice with distilled water followed by soaking in 10 mL of 10 M HCl for 16 h. After incubation, the sample was diluted with 40 mL of distilled water and an acid hydrolysis was followed by autoclaving at 130 °C for 2 h. The suspension was neutralized with 10 M NaOH. A 1 mL portion of the sample was mixed with 1 mL of acetylacetone reagent and incubated for 20 min in a boiling water bath. After the sample was cooled, 6 mL of ethanol and 1 mL of Ehrlich reagent were added and heated at 65 °C for 10 min. *N*-Acetylglucosamine was measured from the optical density at 530 nm against a reagent blank.^34^

#### Chemical Composition Analysis

Before and after fermentation, the chemical constituents of the SCB treated under the optimal conditions and untreated SCB fibers were measured according to the Technical Association of the Pulp and Paper Industry (TAPPI) standard method. The lignin content was analyzed according to TAPPI standard T222 om-98, and the lignin content was calculated using eq 4:
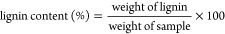
4

The holocellulose constituent was analyzed according to the acid chlorite method.^35^ The holocellulose content was calculated using the eq 5:
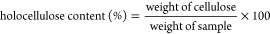
5

The α-cellulose content was estimated according to the TAPPI T202 om-88 method. The content of α-cellulose was calculated using eq 6.^36^ An average of three replicates was calculated for each sample. The α-cellulose content was subtracted from the holocellulose constituents to obtain the amount of hemicellulose:^36^
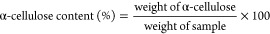
6

#### Physical Characterization

The structural modifications of native and pretreated SCB were observed using an SEM instrument (Hitachi, Jeol JSM-5600 LV, Japan) with an accelerating voltage of 10 kV. The samples were attached to aluminum stubs and coated under vacuum with gold. The SEM images were captured with magnifications ranging from 100× to 1000×.

## Supporting Information Available

Contour plot graph for the influences of the ultrasonic pretreatment factor on the yield of lovastatin and diagnostics and adequacy of the model on fungal biomass productivity (PDF)
