# Supplementary material for: Ultrasonic Irradiation Enables Facile Production of Lovastatin from Sugar Cane Bagasse
Source: ACS Omega. 2022 Apr 12;7(16):13455–64. doi: 10.1021/acsomega.1c06221 (PMC9088786; doi:10.1021/acsomega.1c06221)
Supplement: Supplementary file 1 — ao1c06221_si_001.pdf [file ao1c06221_si_001.pdf]

Supplementary Information

For

Ultrasonic irradiation enables facile production of lovastatin from sugarcane bagasse

Prapassorn Rugthaworn[a,b], Udomlak Sukatta[b], Prakit Sukyai[a,c]*

[a] Biotechnology of Biopolymers and Bioactive Compounds Special Research Unit, Department of Biotechnology, Faculty of Agro-Industry, Kasetsart University, Chatuchak, Bangkok 10900, Thailand

[b] Kasetsart Agricultural and Agro-Industrial Product Improvement Institute (KAPI), Kasetsart University, Bangkok 10900, Thailand

[c] Center for Advanced Studies for Agriculture and Food, Kasetsart University Institute for Advanced Studies, Kasetsart University, Chatuchak, Bangkok, 10900, Thailand

***Corresponding author**

Address: Biotechnology of Biopolymers and Bioactive Compounds Special Research Unit, Department of Biotechnology, Faculty of Agro-Industry, Kasetsart University, Chatuchak, Bangkok 10900, Thailand.

E-mail address: fagipks@ku.ac.th

Supporting Information: 2 pages including Text and 3 Figures.

Figure S1.

Figure S2.

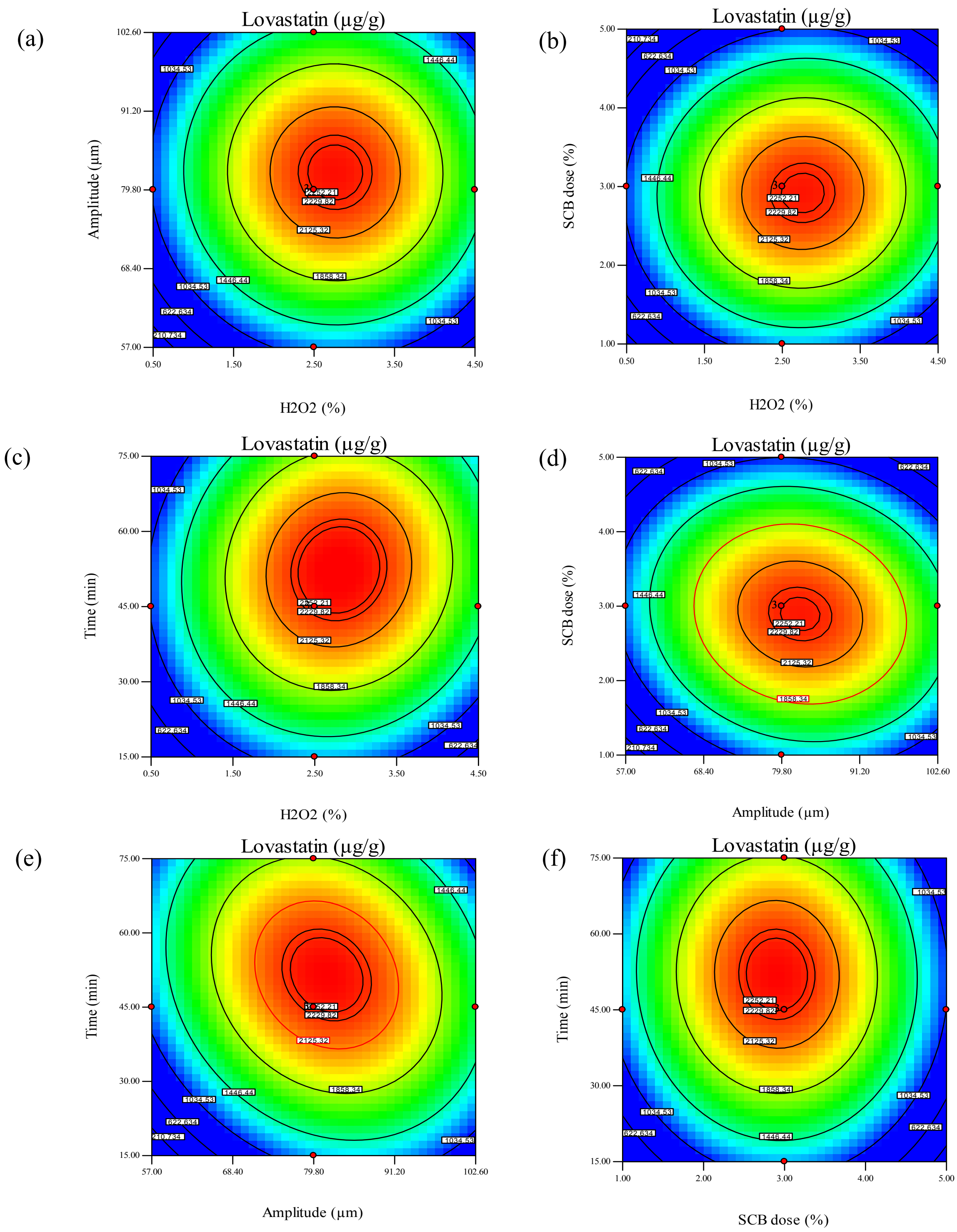

Figure S1. Contour plots graph for the effects of the (a) H_2O_2 concentration (%) versus amplitude (µm), (b) H_2O_2 concentration (%) versus SCB dosage (%), (c) H_2O_2 concentration (%) versus reaction time (min), (d) amplitude (µm) versus SCB dosage (%), (e) reaction time (min) versus amplitude (µm), and (f) SCB dosage (%) versus reaction time (min) on the yield of lovastatin.

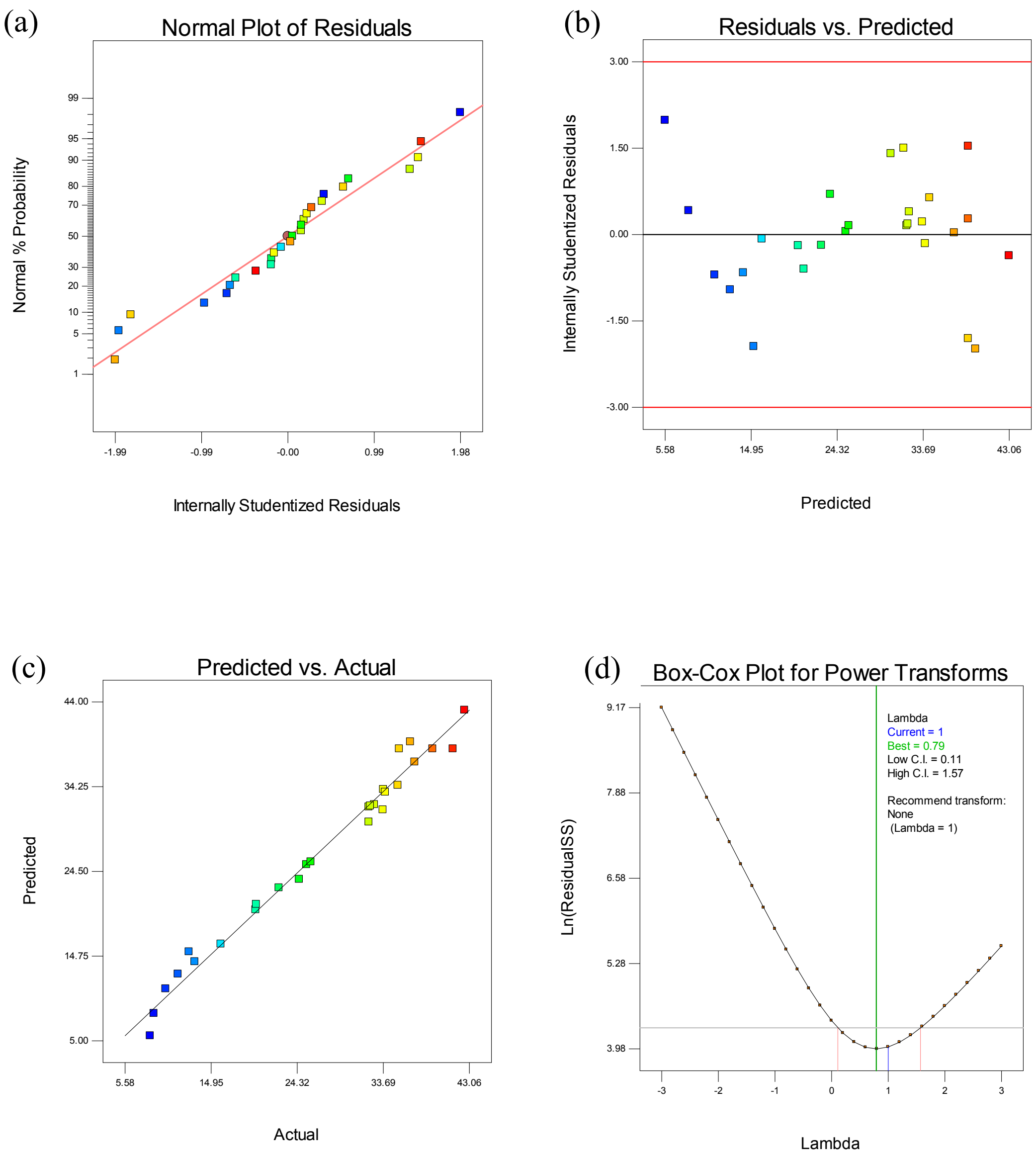

Figure S2. Diagnostics and adequacy of the model on fungal biomass productivity shown by (a) normal probability plot of studentized residuals, (b) plot of internally studentized residuals vs. predicted response, (c) diagnostic plot of the model precision, and (d) BoxCox plot of model transformation.
